# Application value of high-resolution CT imaging features combined with texture analysis in patients with solitary pulmonary nodules

**DOI:** 10.3389/fonc.2026.1942641

**Published:** 2026-09-18

**Authors:** Jie Zong, Haiyan Li, Zhiling Li

**Affiliations:** 1 Department of Radiology, Huabei Petroleum Administration Bureau General Hospital, Renqiu, China; 2 Department of Radiology, Qianxi County People’s Hospital, Qianxi, China; 3 Department of General Surgery, Huabei Petroleum Administration Bureau General Hospital, Renqiu, China

**Keywords:** diagnostic efficacy, entropy, high-resolution computed tomography, solitary pulmonary nodule, texture analysis

## Abstract

**Introduction:**

This retrospective study evaluated whether high-resolution CT (HRCT) texture analysis adds diagnostic value to conventional morphological assessment for differentiating benign from malignant solitary pulmonary nodules (SPNs).

**Methods:**

We analyzed 200 patients with pathologically confirmed SPNs (43 benign, 157 malignant) examined between September 2022 and October 2023, using the most recent preoperative HRCT obtained within one month of confirmation. Conventional CT features and texture parameters (entropy, difference in entropy, sum of entropy) were compared between groups. All continuous predictors were Z-score standardized, and three multivariable logistic-regression models—conventional morphological, texture-only, and combined—were constructed; diagnostic performance was assessed by ROC analysis with optimism-corrected bootstrap internal validation.

**Results:**

The three entropy-related parameters were significantly higher in malignant nodules (P < 0.001). The combined model achieved the highest discrimination (AUC = 0.958; 95% CI, 0.931–0.986; sensitivity 96.8%, specificity 81.4%), significantly outperforming the morphological model alone (DeLong P < 0.001). Multicollinearity was negligible (all predictor variance inflation factors < 1.25), and the optimism-corrected AUC remained high (0.946), supporting favorable internal discrimination after optimism correction.

**Discussion:**

HRCT-based entropy parameters provided incremental information beyond morphology within this cohort; independent external validation is required before clinical application.

## 1 Introduction

Solitary pulmonary nodules (SPNs) are common clinical findings, defined as rounded opacities measuring 3 cm or less in diameter that are completely surrounded by aerated lung parenchyma (1). Epidemiological data indicate that approximately 1%–12% of SPNs are ultimately malignant, and surgical resection at this early stage can yield a high long-term survival rate (2, 3). Consequently, early and accurate differential diagnosis is of substantial importance for guiding clinical management and improving patient prognosis.

With the ongoing advancement of medical imaging, computed tomography (CT) has become a cornerstone of clinical practice. Conventional CT imaging, which relies on the observation of morphological and density characteristics, holds established value in differentiating benign from malignant SPNs (4). Conventional morphological signs such as lobulation, spiculation, and pleural indentation, as standardized in the Fleischner Society glossary and management guidelines (5), remain central to radiological nodule assessment. However, diagnosis based solely on conventional CT features can be challenging in many cases. Computerized texture analysis addresses this limitation by quantifying the distribution, patterns, and interrelationships of pixel gray levels within a lesion, thereby extracting information imperceptible to the human eye and reflecting underlying tissue heterogeneity (6, 7). As a non-invasive and easy-to-implement technique, it represents a promising auxiliary diagnostic tool. High-resolution computed tomography (HRCT) provides enhanced visualization of fine anatomical detail and internal nodule architecture, offering superior morphological and structural information that aids in early identification (8).

Texture analysis, as a core component of radiomics, has been an active research focus in thoracic imaging for over a decade. Early work demonstrated that gray-level co-occurrence matrix (GLCM) features—particularly entropy and sum entropy—differ significantly between benign and malignant pulmonary nodules (9, 10), and subsequent studies incorporated such features into logistic-regression and nomogram models to support malignancy prediction (11, 12). However, two gaps persist. First, reported diagnostic performance varies widely across studies, partly because texture features are sensitive to acquisition and reconstruction settings and because many studies lack rigorous reproducibility assessment (1, 13). Second, relatively few studies have directly quantified the incremental value gained by adding entropy-related texture parameters to routine, clinically interpretable morphological signs within a single, pathologically confirmed cohort. The present study addresses these gaps by restricting the model to a small, reproducible set of entropy-related parameters that demonstrated acceptable inter-observer reproducibility, and by explicitly comparing a conventional morphological model, a texture-only model, and a combined model in the same cohort, thereby isolating the incremental diagnostic contribution of texture analysis.

Despite these advancements, the characterization of nodules with atypical manifestations remains challenging when relying exclusively on radiological signs. Constructing a comprehensive diagnostic model that integrates quantitative texture parameters with conventional visual assessment may effectively bridge this diagnostic gap. This study therefore aims to evaluate the incremental diagnostic value of combining HRCT-based texture analysis with conventional morphological features for the accurate differentiation of benign from malignant SPNs. By quantifying the incremental value of a parsimonious entropy-based model over routine morphological assessment, this study seeks to evaluate its potential as an interpretable adjunct to conventional CT assessment.

## 2 Materials and methods

### 2.1 Patient selection

A total of 200 patients with SPNs treated between September 2022 and October 2023 were retrospectively included (**Figure 1**). Cases were selected (not consecutively enrolled) on the basis of the availability of diagnostic-quality preoperative HRCT images and complete pathological records. Some patients underwent more than one CT examination during follow-up; for each patient, only the most recent preoperative examination obtained before pathological confirmation was used for quantitative analysis.

**Figure 1:**
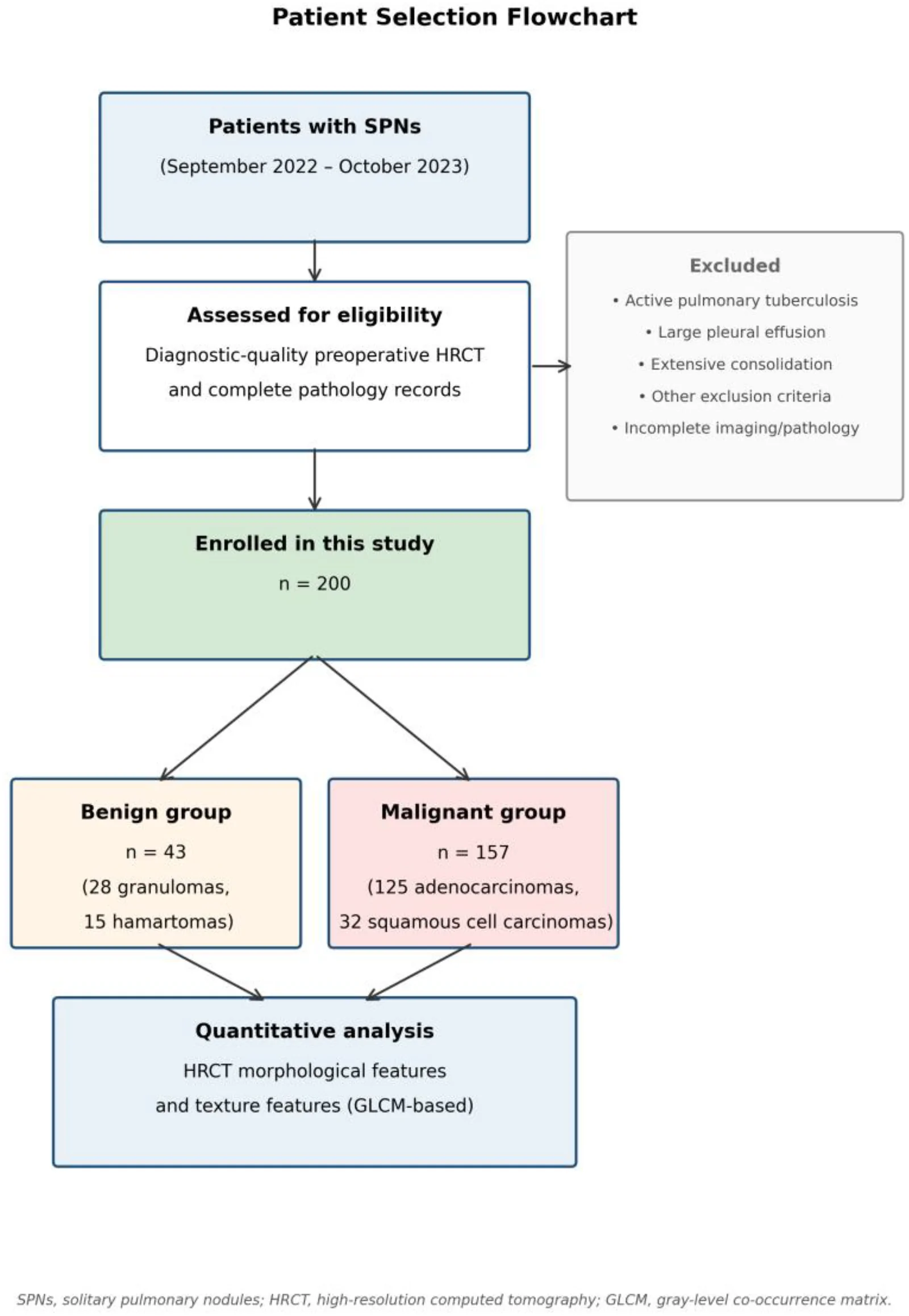
Flowchart of patient selection. From patients with pathologically confirmed SPNs during the study period (selected, non-consecutive), cases were excluded according to the prespecified criteria, including active pulmonary tuberculosis, large pleural effusion causing lung compression, extensive consolidation precluding reliable nodule delineation, and incomplete imaging/pathology data; other exclusion criteria are detailed in the Methods. A total of 200 patients were included and classified by histopathology into a benign group (n = 43; 28 granulomas, 15 hamartomas) and a malignant group (n = 157; 125 adenocarcinomas, 32 squamous cell carcinomas).

### 2.2 CT acquisition

CT images were acquired on three multidetector CT scanners: UIH uCT550 (n = 52), GE Revolution CT (n = 85), and GE LightSpeed VCT (n = 63). All examinations were non-contrast scans performed in the supine position during end-inspiration breath-hold, covering the lung apices to the costophrenic angles. Typical parameters were: tube voltage 120 kVp, automatic tube-current modulation, collimation ≤1.25 mm, pitch ~1.0–1.2, rotation time ~0.5–1.2 s, and reconstruction slice thickness/interval ≤1.25 mm, with a 512×512 matrix. Images were reconstructed using both lung (sharp) and soft-tissue (standard) kernels and reviewed on lung- and mediastinal-window settings. Before texture-feature extraction, all nodule volumes were resampled to isotropic 1.0 × 1.0 × 1.0-mm voxels to standardize voxel geometry. This resampling step does not fully eliminate variability related to scanner type or reconstruction kernel. Scanner type was not included as a covariate in the diagnostic models, and no formal scanner-specific confounding analysis was performed. These thin-section reconstructions are referred to as HRCT images hereafter.

### 2.3 Radiation dose reference

For radiation context, volume CT dose index (CTDIvol) and dose-length product (DLP) were recorded from the scanner dose reports. For reference, published adult diagnostic chest CT protocols report a CTDIvol of approximately 9–12 mGy and a DLP of approximately 334–443 mGy·cm (effective dose ~4.7–6.2 mSv using DLP × 0.014) (14); these are cited as contextual literature values and are not outcomes of the present study.

### 2.4 Reference standard

Clinicopathological findings served as the diagnostic gold standard, with benign/malignant outcomes defined by the final histopathological diagnosis. Pathological confirmation was based on surgical resection and/or biopsy specimens (CT-guided percutaneous transthoracic needle biopsy or bronchoscopic biopsy). The decision between biopsy and resection considered nodule size, imaging suspicion (e.g., spiculation/lobulation, pleural indentation), interval growth when available, patient risk factors, and multidisciplinary discussion. Patients managed by CT surveillance alone without pathological confirmation were not included.

### 2.5 Inclusion and exclusion criteria

Inclusion criteria: (1) an initial imaging diagnosis of SPN (incidental/asymptomatic or symptomatic); (2) nodule diameter 0.5–3.0 cm; (3) age 20–80 years; (4) no prior treatment of the nodule before admission; and (5) availability of diagnostic-quality HRCT obtained within 1 month before pathological confirmation.

Exclusion criteria: (1) active malignancy or any history of malignant tumor within the past 5 years; (2) other pulmonary space-occupying lesions; (3) active pulmonary tuberculosis; (4) large pleural effusion causing substantial lung compression; (5) extensive pulmonary consolidation in which a solitary nodule could not be reliably delineated; (6) severe cardiac, cerebral, hepatic, or renal dysfunction, or metabolic disease; (7) autoimmune deficiency or hematological disease; (8) severe neuropsychiatric conditions or poor compliance; and (9) poor-quality CT with significant artifacts, or nondiagnostic pathology without subsequent confirmation. Pulmonary fibrosis was not an exclusion criterion, and such patients were included.

### 2.6 Morphological feature assessment

HRCT scans were evaluated for specific morphological features. Lobulation, spiculation, pleural indentation, and the air-bronchogram sign were recorded as features suggestive of malignancy, whereas a cavity or benign-pattern calcification was recorded as suggestive of benignity (5, 15). Calcification, cavitation, and the air-bronchogram sign were recorded qualitatively to aid characterization; their per-case frequencies were not systematically tabulated, and these signs were not entered into the diagnostic models. Although histopathology served as the gold standard, these conventional features were assessed to reflect routine radiological evaluation and to provide an interpretable baseline for comparison with the quantitative texture results.

### 2.7 Segmentation and texture feature extraction

Quantitative texture features—including entropy, sum of entropy, difference in entropy, contrast, correlation, and sum of averages—were extracted from the nodule regions. All images were imported into ITK-SNAP (version 4.2.0, www.itksnap.org) for manual segmentation of three-dimensional (3D) regions of interest (ROIs); the entire volume of each nodule was contoured slice-by-slice on axial lung-window images (**Figure 2**). Primary segmentation was performed by a board-certified radiologist with 10 years of thoracic imaging experience, blinded to the pathological results. Texture features were subsequently extracted from the segmented 3D ROIs using RIAS (version 1.0.0; https://github.com/lisherlock/RMIT) (16), following resampling to isotropic 1.0 × 1.0 × 1.0 mm³ voxels. The reported parameters were GLCM-derived texture features.

**Figure 2:**
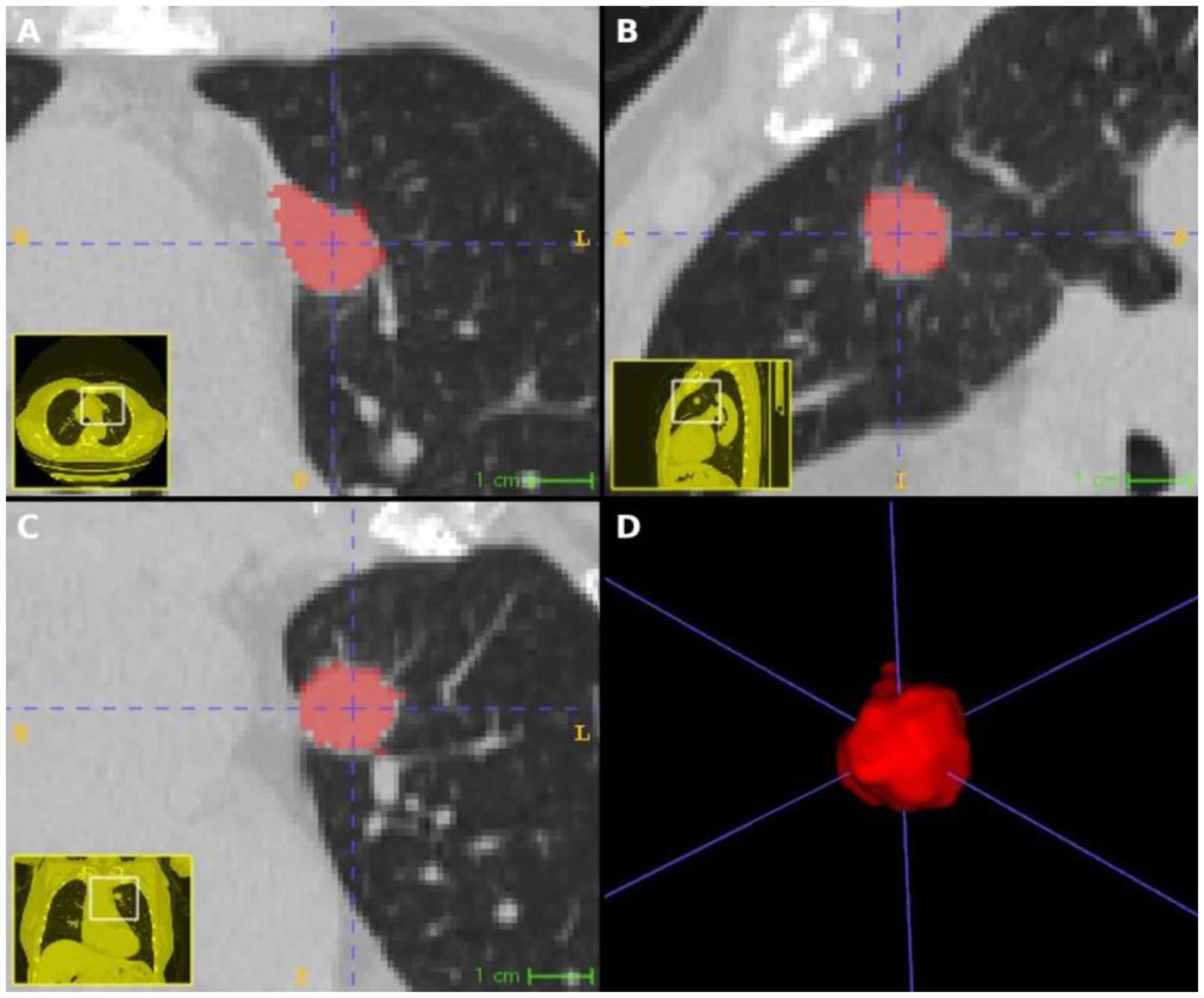
Representative three-dimensional ROI segmentation using ITK-SNAP. The nodule (red overlay) was manually contoured slice-by-slice on **(A)** axial, **(B)** sagittal, and **(C)** coronal lung-window images; **(D)** shows the corresponding three-dimensional reconstruction of the segmented nodule volume. Scale bar = 1 cm.

### 2.8 Inter-observer reliability

To evaluate inter-observer reliability, 30 cases were randomly selected and independently segmented by a second board-certified radiologist (10 years of experience) in a blinded manner. Inter-observer reproducibility was assessed using the intraclass correlation coefficient (ICC) for each extracted feature, with ICC > 0.75 considered robust (17).

### 2.9 Feature interpretation

Briefly, entropy reflects the randomness/complexity of the gray-level distribution and serves as a surrogate of intranodular heterogeneity; difference in entropy and sum of entropy quantify heterogeneity based on gray-level differences and sums in the co-occurrence statistics; contrast reflects local intensity variation; and correlation reflects the linear dependency of gray levels across neighboring pixels (18).

### 2.10 Statistical analysis

Primary statistical analyses were performed using SPSS (version 27.0); multicollinearity assessment and optimism-corrected bootstrap internal validation were additionally performed in Python using statsmodels. Normally distributed continuous data were expressed as mean ± standard deviation. Homogeneity of variance was assessed using Levene’s test. The independent-samples Student’s t-test was used when the assumption of equal variances was met (Levene P ≥ 0.05); otherwise, Welch’s t-test was applied. Categorical data were presented as numbers and percentages and analyzed with the chi-square test, with continuity correction applied for 2 × 2 tables. Available baseline variables, including sex, age, BMI, nodule area, and upper-lobe location, were evaluated between the benign and malignant groups. Upper-lobe location was defined as a nodule located in either the right or left upper lobe. Smoking exposure and other relevant historical clinical risk factors were incompletely documented in the retrospective records and were therefore not included in model development. No missing values were present among the variables included in the statistical analyses; therefore, no imputation was performed. Candidate variables evaluated for possible model entry included the available baseline variables (sex, age, BMI, nodule area, and upper-lobe location), three conventional morphological signs (lobulation, spiculation, and pleural indentation), and six GLCM-derived texture features (entropy, difference in entropy, sum of entropy, contrast, correlation, and sum of averages). Variables with P < 0.05 in univariate analyses were considered for model entry. This screening retained the three morphological signs and three entropy-related texture features (entropy, difference in entropy, and sum of entropy); the retained texture features also demonstrated ICC values > 0.75 in the reproducibility subset. The selected predictors were subsequently entered simultaneously using the forced-entry (Enter) method to construct the morphological, texture-only, and combined logistic-regression models; no automated stepwise selection was performed within the multivariable models. A two-tailed P < 0.05 was considered statistically significant.

Multivariable binary logistic regression with the forced-entry (Enter) method was used to construct three diagnostic models: a conventional morphological model (lobulation, spiculation, pleural indentation); a texture-only model (entropy, difference in entropy, sum of entropy); and a combined model incorporating all six predictors. Because the texture predictors had narrow numerical ranges, all continuous predictors were Z-score standardized prior to logistic regression to yield interpretable coefficients; as a monotonic linear transformation, standardization does not alter model discrimination. With the pathological outcome as the dependent variable (benign = 0, malignant = 1), the texture model yielded: Logit(P) = 2.386 + 1.245 × z(Entropy) + 1.064 × z(Difference_in_Entropy) + 1.482 × z(Sum_of_Entropy).

Multicollinearity was assessed for each model using the variance inflation factor (VIF); all predictor VIFs were below 1.25, indicating no meaningful collinearity among predictors. Predicted probabilities were used as test variables in receiver operating characteristic (ROC) analysis to determine the area under the curve (AUC); the Youden index defined optimal cut-offs with corresponding sensitivity and specificity. The 95% confidence intervals for all AUC estimates were calculated using the DeLong method, and DeLong’s test was used for pairwise comparison of AUCs (19). To assess potential overfitting given the limited sample size, internal validation was performed using Harrell’s optimism-corrected bootstrap (1000 resamples), reporting apparent performance, optimism, and the optimism-corrected AUC for each model. This internal-validation approach is in line with established recommendations for clinical prediction modeling, including the use of bootstrap optimism correction as recommended in the TRIPOD+AI reporting guideline (20). No formal *a priori* sample-size calculation was performed because this retrospective analysis was based on a fixed dataset of 200 selected, pathologically confirmed cases meeting the study criteria. The combined model contained six predictor parameters, while the smaller outcome group comprised 43 benign cases, corresponding to approximately 7.2 observations in the smaller outcome category per predictor parameter. This limited effective sample size may increase uncertainty in model estimation and the risk of optimism. Bootstrap internal validation was therefore used to estimate and correct optimism in model discrimination; however, this procedure does not compensate for the limited sample size, and the model requires validation in larger independent cohorts.

## 3 Results

### 3.1 Baseline characteristics

The baseline characteristics are summarized in **Table 1**. No statistically significant differences were observed in sex, age, body mass index (BMI), nodule area, or upper-lobe location between the benign and malignant groups (all P > 0.05). Although upper-lobe location was more frequent among malignant than benign nodules (57.3% vs. 39.5%), the difference did not reach statistical significance (χ² = 3.609, P = 0.057). The benign group comprised 43 patients (mean age 60.70 ± 8.37 years), including 28 inflammatory granulomas and 15 pulmonary hamartomas; the malignant group comprised 157 patients (mean age 60.51 ± 7.75 years), including 125 adenocarcinomas and 32 squamous cell carcinomas. Overall, 145 cases were confirmed by surgical resection and 55 by biopsy. Detailed values are provided in **Table 1**.

**Table 1:** Comparison of baseline data of patients with SPNs.

Characteristic	Benign group (n=43)	Malignant group (n=157)	*χ^2^/t*	*P*
Sex			0.000	1.000
Male	26 (60.47%)	95 (60.51%)		
Female	17 (39.53%)	62 (39.49%)		
Age (years)	60.70 ± 8.37	60.51 ± 7.75	0.139	0.890
BMI	28.02 ± 3.68	27.20 ± 3.08	1.471	0.143
Nodule area (cm^2^)	2.34 ± 0.53	2.51 ± 0.55	-1.69	0.093
Upper-lobe location	17 (39.53%)	90 (57.32%)	3.609	0.057
**Pathological confirmation**			–	–
Surgical resection	30 (69.77%)	115 (73.25%)		
Biopsy	13 (30.23%)	42 (26.75%)		
**Histological subtype**			–	–
Inflammatory granuloma	28 (65.12%)	0 (0%)		
Pulmonary hamartoma	15 (34.88%)	0 (0%)		
Adenocarcinoma	0 (0%)	125 (79.62%)		
Squamous cell carcinoma	0 (0%)	32 (20.38%)		

BMI, body mass index. Data are presented as mean ± SD or n (%).

Bold text indicates category headings and does not denote statistical significance.

### 3.2 Inter-observer reproducibility

The inter-observer reproducibility of the extracted texture features was high. The three key texture parameters retained for analysis—entropy, difference in entropy, and sum of entropy—all achieved an intraclass correlation coefficient (ICC) greater than 0.75, supporting their robustness for subsequent modeling.

### 3.3 HRCT and texture findings

Representative HRCT images of the four nodule types are shown in **Figures 3A–D**; the detailed longitudinal description of the granuloma case is provided in the figure legend. Regarding texture and morphological findings, entropy was significantly higher in the malignant group (1.52 ± 0.08) than in the benign group (1.45 ± 0.07; P < 0.001); difference in entropy was higher in the malignant group (0.98 ± 0.07 vs. 0.91 ± 0.05; P < 0.001); and sum of entropy was higher in the malignant group (1.14 ± 0.02 vs. 1.11 ± 0.03; P < 0.001). Lobulation, spiculation, and pleural indentation were significantly more frequent in the malignant group (all P < 0.05). No significant differences were found for maximum diameter, mean CT value, sum of averages, correlation, or contrast (P > 0.05). All compared parameters are detailed in **Table 2**.

**Figure 3:**
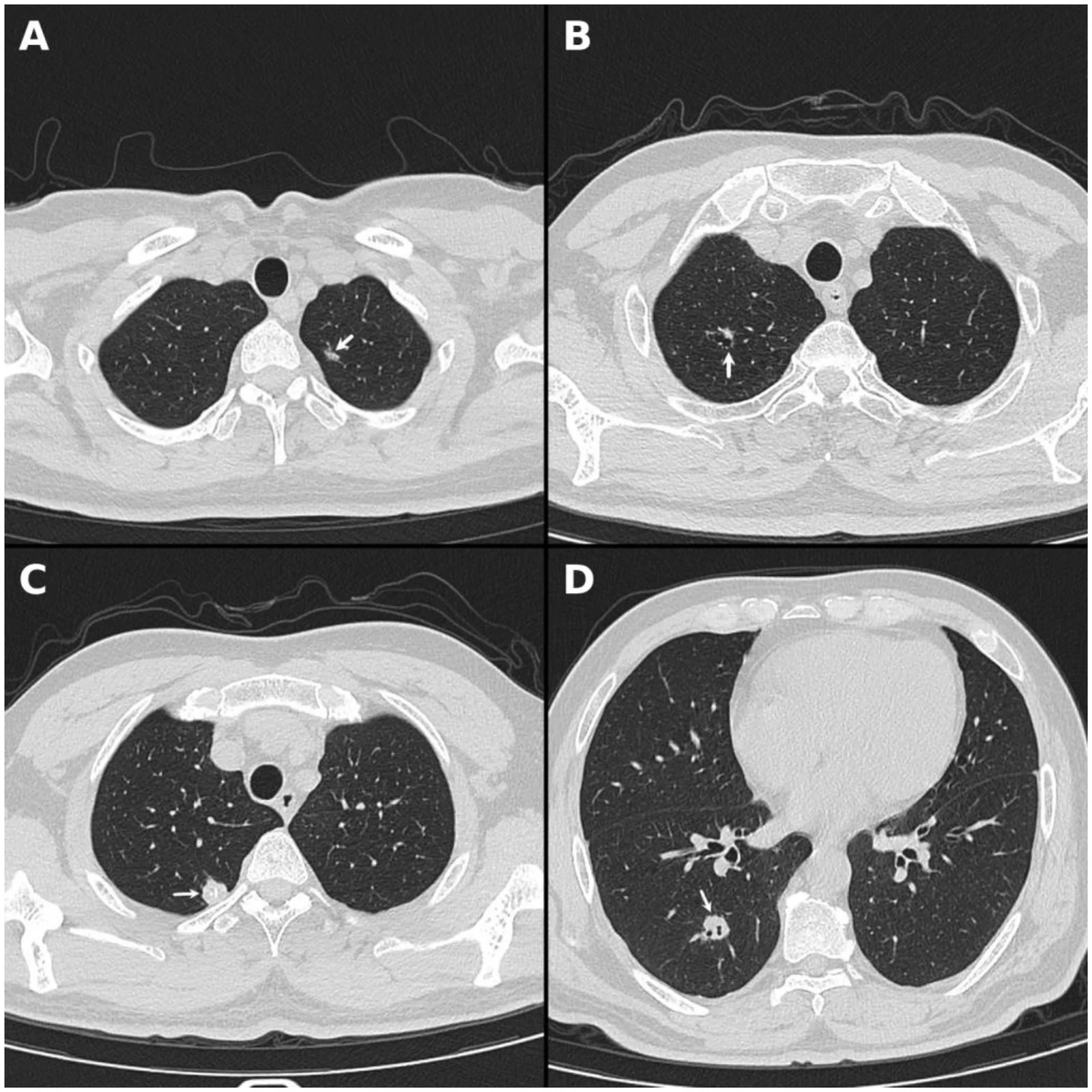
Representative HRCT images of solitary pulmonary nodules (lung window). **(A)** Inflammatory granuloma on follow-up, showing increased attenuation, reduced volume, and well-defined smooth margins, consistent with a subsolid nodule (arrow). The follow-up image is shown for illustrative purposes only; for each patient, a single preoperative CT examination was used for texture analysis. **(B)** Cystic adenocarcinoma presenting as a subsolid nodule with a cystic component and adjacent vessel convergence (arrow). **(C)** Pulmonary hamartoma with smooth margins, shallow lobulation, and internal popcorn-like calcification visible on the optimized lung-window setting (arrow). **(D)** Early-stage squamous cell carcinoma appearing as a solid nodule with shallow lobulation and a vessel traversing the lesion (arrow).

**Table 2:** High-resolution CT imaging findings.

Parameters	Benign group (n = 43)	Malignant group (n = 157)	Statistic (t/χ^2^)	P value
Conventional CT parameters				
Maximum diameter (cm)	2.10 ± 0.23	2.12 ± 0.33	-0.487	0.627
Mean CT value (HU)	25.03 ± 7.55	23.84 ± 6.91	0.98	0.328
Morphological signs				
Lobulation	19 (44.19%)	111 (70.70%)	9.298	0.002
Spiculation	14 (32.56%)	122 (77.71%)	29.580	<0.001
Pleural indentation	8 (18.60%)	95 (60.51%)	22.083	<0.001
Texture parameters				
Entropy	1.45 ± 0.07	1.52 ± 0.08	-5.551	<0.001
Difference in entropy	0.91 ± 0.05	0.98 ± 0.07	-6.235	<0.001
Sum of entropy	1.11 ± 0.03	1.14 ± 0.02	-7.577	<0.001
Sum of averages	68.43 ± 3.09	68.04 ± 3.54	0.64	0.523
Correlation	-0.13 ± 0.03	-0.12 ± 0.03	-1.684	0.094
Contrast	207.23 ± 23.45	210.04 ± 20.57	-0.772	0.441

Continuous data are presented as mean ± standard deviation. Homogeneity of variance was assessed using Levene’s test; the independent-samples Student’s t-test was used when Levene P ≥ 0.05, and Welch’s t-test was used when Levene P < 0.05. Categorical data are presented as n (%), and comparisons were performed using the chi-square test, with continuity correction applied for 2 × 2 tables.

HRCT, high-resolution computed tomography; CT, computed tomography; HU, Hounsfield units.

### 3.4 Diagnostic performance of the models

Among individual texture parameters, entropy yielded an AUC of 0.764; the AUCs for difference in entropy and sum of entropy alone were 0.758 and 0.814, respectively. The texture-only model (entropy + difference in entropy + sum of entropy) achieved an AUC of 0.914, and the conventional morphological model (lobulation, spiculation, pleural indentation) an AUC of 0.861. The combined model achieved the highest AUC of 0.958 (95% CI: 0.931–0.986), with a sensitivity of 96.8% (95% CI, 92.8–98.6) and a specificity of 81.4% (95% CI, 67.4–90.3). The full combined-model equation was Logit(P) = −0.559 + 1.418 × z(Entropy) + 1.173 × z(Difference_in_Entropy) + 1.216 × z(Sum_of_Entropy) + 1.244 × Lobulation + 2.219 × Spiculation + 2.377 × Pleural_indentation, in which all six predictors remained independently significant (odds ratios 3.2–10.8; all P ≤ 0.043); the training-cohort standardization parameters (mean ± SD) were entropy 1.500 ± 0.079, difference in entropy 0.965 ± 0.075, and sum of entropy 1.132 ± 0.024. At the Youden-optimal threshold, the positive and negative predictive values were 95.0% (95% CI, 90.4–97.4) and 87.5% (95% CI, 73.9–94.5) for the combined model, 93.9% and 51.5% for the morphological model, and 95.3% and 69.2% for the texture-only model. The DeLong test confirmed that the combined model significantly outperformed the morphological model alone (P < 0.001) (**Table 3**; **Figure 4**). Multicollinearity was negligible in all three models (all predictor VIFs < 1.25; texture predictors ≈ 1.02). Internal bootstrap validation indicated limited optimism in discrimination for the final model specifications (0.007–0.012), with optimism-corrected AUCs of 0.850, 0.907, and 0.946 for the morphological, texture-only, and combined models, respectively (**Table 3**; **Figure 4**).

**Table 3:** Diagnostic efficacy of individual parameters and combined models in differentiating benign and malignant SPNs.

Parameter/diagnostic model	AUC	95% CI	Cut-off value	Sensitivity (%)	Specificity (%)
Ent	0.764	0.689–0.839	1.485	70.70	76.7
DEnt	0.758	0.689–0.827	0.965	60.5	86.0
SEnt	0.814	0.729–0.899	1.115	86.6	65.1
Texture-only model (Ent + DEnt + SEnt)	0.914	0.862–0.966	0.692	89.8	83.7
Conventional morphological model	0.861	0.803–0.919	0.891	79.0	81.4
Morphology-texture combined model	0.958^a,b^	0.931–0.986	0.524	96.8	81.4

AUC, area under the curve; CI, confidence interval; Ent, entropy; DEnt, difference in entropy; SEnt, sum of entropy. Cut-off values were determined by ROC analysis using the Youden index; for the logistic-regression models, the reported cut-offs represent predicted-probability thresholds. The conventional morphological model incorporates lobulation, spiculation, and pleural indentation. The morphology-texture combined model incorporates the three morphological signs and the three significant texture parameters. ^a^ *P* < 0.001 compared with the conventional morphological model (DeLong’s test). ^b^ *P* = 0.020 compared with the texture-only model (DeLong’s test). There was no statistically significant difference between the texture-only model and the conventional morphological model (*P* = 0.159).

**Figure 4:**
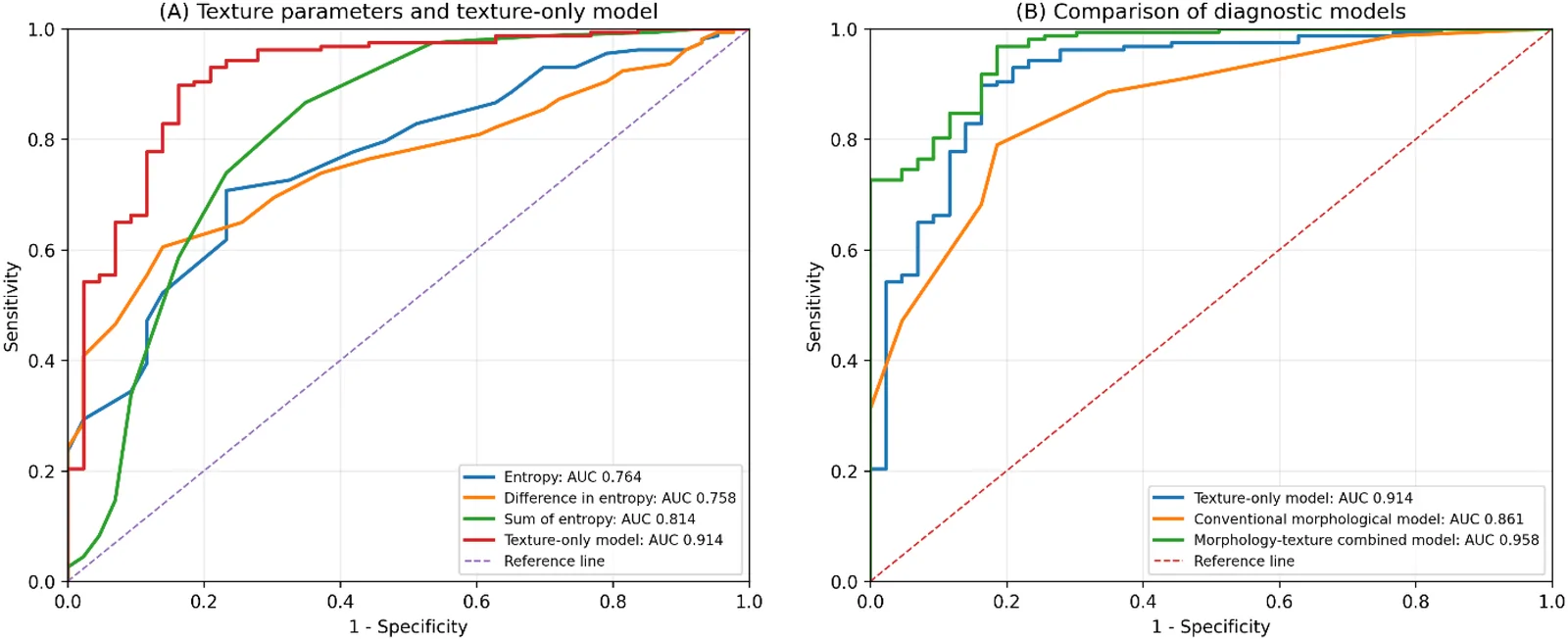
ROC curves for differentiating benign from malignant SPNs. **(A)** ROC curves for individual texture parameters (entropy, difference in entropy, sum of entropy) and the texture-only model (AUC = 0.914). **(B)** Comparative ROC analysis: the morphology-texture combined model achieved the highest AUC (0.958), significantly superior to the conventional morphological model (AUC = 0.861; DeLong P < 0.001).

## 4 Discussion

This study examined whether quantitative texture analysis adds diagnostic value beyond conventional morphological assessment for differentiating benign from malignant SPNs. Entropy-related texture parameters were independently associated with malignancy, and their combination with routine morphological signs yielded the best-performing model in this cohort.

Differentiating benign from malignant SPNs remains challenging because inflammatory nodules can closely mimic malignancy on CT. Tumor heterogeneity associated with malignancy may not be fully captured by conventional visual assessment (6, 21). In this cohort, mean CT attenuation did not differ significantly between groups, whereas entropy, difference in entropy, and sum of entropy were significantly higher in malignant nodules (all P < 0.001). These findings suggest greater intratumoral heterogeneity in malignant nodules, reflected by a more disordered distribution of gray-level intensities on CT. Entropy-related measures quantify this complexity and may capture heterogeneity not readily apparent on visual assessment, thereby complementing conventional morphological assessment.

Our results are consistent with previous radiomic studies of pulmonary nodules. Wang et al. reported differences in entropy-related texture features between benign and malignant nodules (10), while Dennie et al. showed that CT texture analysis could help distinguish primary lung cancer from granulomatous nodules (9). The latter is particularly relevant to our cohort because granulomas constituted the main benign subgroup. Texture-based discrimination may become more difficult when benign lesions are themselves heterogeneous. In a histologically confirmed series of 175 patients, conventional texture parameters showed limited accuracy for distinguishing benign from malignant lung lesions, partly because inflammatory lesions such as granulomas may share heterogeneous imaging characteristics with malignancy (11). This finding cautions against relying on texture features alone.

In our study, individual texture features showed only moderate discrimination (AUCs, 0.758–0.814), whereas the texture-only model achieved an AUC of 0.914. The morphology-texture combined model achieved an AUC of 0.958 and significantly outperformed the morphological model alone (AUC 0.861; DeLong P < 0.001). Previous work has also explored models combining CT texture features with non-imaging information, including urine energy-metabolism measures (12). Although the variables used in that study differed from ours, both studies support evaluating quantitative texture information together with other sources of diagnostic information rather than in isolation.

While such conventional signs as lobulation and spiculation remain the cornerstone of radiological diagnosis, they have inherent limitations (morphological model AUC = 0.861). Notably, integrating entropy-related texture parameters with these morphological features improved diagnostic performance to an AUC of 0.958; this significant improvement (DeLong P < 0.001) suggests that sub-visual texture analysis provides incremental value, helping bridge the gap left by routine visual inspection. Reconstruction kernel is an important technical determinant of texture quantification: sharp (lung) kernels enhance edge detail but increase noise, whereas soft-tissue (standard) kernels yield smoother images, and these differences can substantially alter absolute texture values. Prior work has shown that radiomic—especially texture—features are poorly reproducible across different kernels (13), and that kernel, slice thickness, and contrast enhancement all affect pulmonary-nodule radiomic signatures (1). We mitigated this by restricting analysis to thin-section reconstructions and resampling all nodules to isotropic 1.0-mm voxels; nonetheless, residual kernel-related variability remains. Qualitative assessment and quantitative radiomics are therefore best regarded as complementary: established morphologic predictors provide important diagnostic clues that may not be fully encoded by texture metrics, whereas radiomics provides quantitative sub-visual information; their integration may enable a more comprehensive characterization of nodule features.

The morphology-texture model should be considered an adjunct to guideline-based nodule management rather than a replacement for it. Current pathways use nodule size, morphology, interval change, and patient-level risk factors to guide surveillance or further diagnostic evaluation (5). In this selected, pathologically confirmed cohort, texture features improved discrimination beyond CT morphology. However, we did not compare the model with a validated clinical-radiological risk model or a guideline-based management strategy, nor did we assess its effect on clinical decision-making. Entropy-based texture analysis may therefore complement morphological assessment in this setting, but its added clinical value beyond current risk-stratification pathways remains unproven. Prospective studies should determine whether adding texture information to clinical-radiological assessment improves management decisions, reduces unnecessary invasive procedures, or improves patient outcomes before clinical implementation.

In clinical practice, pulmonary nodule assessment is often longitudinal, whereas our texture analysis used only one preoperative CT examination per patient. Serial changes in nodule size, attenuation, morphology, and quantitative imaging features may provide information beyond a single scan. A recent multicenter study reported that longitudinal CT-feature variability improved prediction compared with baseline imaging features alone (22). Future studies should examine whether dynamic texture changes, together with interval morphological changes and established clinical risk factors, improve nodule risk assessment. A retrospective study in patients with fibrosing interstitial lung disease also evaluated pulmonary nodules using longitudinal imaging together with clinical and functional information (23). Such approaches require prospective validation before their clinical value can be established.

This study has several strengths. First, all included SPNs had definitive pathological confirmation, providing a robust reference standard. Second, CT feature assessment and texture-feature extraction followed a standardized workflow, improving internal consistency. Third, the combined model uses a small set of readily computable entropy-related parameters, which may facilitate model interpretation.

Several limitations should be acknowledged. First, this retrospective single-center study included a selected, non-consecutive cohort limited to pathologically confirmed SPNs. Benign nodules managed without tissue sampling and some slow-growing malignant nodules under surveillance were therefore not represented. This pathology-enriched sampling resulted in a high prevalence of malignancy (157/200, 78.5%), far above that expected in unselected populations with incidentally detected pulmonary nodules, and introduced both selection and spectrum bias. Diagnostic performance—especially PPV and NPV, which are prevalence-dependent—should not be extrapolated to routine pulmonary-nodule populations; sensitivity and specificity may also vary when the clinical and disease spectra differ. The small benign subgroup (n = 43) also limited the effective sample size for multivariable modeling and may increase uncertainty in coefficient estimates and model performance. Second, inter-observer reproducibility was assessed in 30 cases; although consistent with common practice, a larger reproducibility sample and multi-reader, multi-platform testing would further strengthen confidence in feature robustness. Third, CT data were acquired using three scanners and both sharp and standard reconstruction kernels, leaving residual technical heterogeneity. Isotropic resampling standardized voxel geometry but could not remove scanner- or kernel-related variability in texture features (1, 13). Scanner type was not included as a covariate, and scanner-specific confounding was not formally assessed; therefore, residual scanner- and kernel-related effects on texture measurements cannot be excluded. Fourth, texture features are sensitive to acquisition settings, reconstruction choices, and ROI delineation. Optimism-corrected bootstrap was used for internal validation; it estimates optimism within the development cohort but does not establish generalizability to independent populations. Both the apparent AUC (0.958) and the optimism-corrected AUC (0.946) were derived from the same selected, single-center cohort. The corrected AUC therefore reflects internally validated discrimination within this cohort rather than externally validated performance. Independent prospective multicenter validation with standardized protocols and more representative populations is needed before clinical implementation. Fifth, we evaluated available baseline variables, including upper-lobe location, but key clinical risk factors—particularly smoking exposure—were incompletely documented and could not be reliably incorporated into model development. Active malignancy or a history of malignancy within the preceding 5 years was excluded by study design. Accordingly, the incremental value demonstrated here is relative to conventional CT morphology, not to a comprehensive or validated clinical-radiological risk model. Future prospective studies should systematically collect established clinical risk factors and evaluate whether texture features add value beyond clinical-radiological risk assessment (2, 5). Formal calibration was not assessed in the present study and should be evaluated in future internal and external validation.

## 5 Conclusions

In conclusion, HRCT-based computerized texture analysis provides a non-invasive and potential complementary approach to conventional imaging, reflecting the internal structural heterogeneity of pulmonary nodules. A combined model integrating entropy, difference in entropy, and sum of entropy with conventional morphological assessment demonstrated incremental diagnostic value within this internally validated cohort. Its generalizability and clinical utility require confirmation in independent prospective multicenter cohorts before clinical implementation.

## Data Availability

The data presented in this study are available from the corresponding author on reasonable request, subject to applicable ethical and privacy requirements. The data are not publicly available due to patient privacy restrictions. The analysis code—Python scripts for standardized logistic-regression modeling, multicollinearity (VIF) assessment, optimism-corrected bootstrap internal validation, and baseline statistics—is available from the corresponding author on reasonable request.

## References

[1] He L Huang Y Ma Z Liang C Liang C Liu Z . Effects of contrast-enhancement, reconstruction slice thickness and convolution kernel on the diagnostic performance of radiomics signature in solitary pulmonary nodule. Sci Rep. (2016) 6:34921. doi: 10.1038/srep34921 27721474 PMC5056507

[2] Wu Z Huang T Zhang S Cheng D Li W Chen B . A prediction model to evaluate the pretest risk of Malignancy in solitary pulmonary nodules: evidence from a large Chinese southwestern population. J Cancer Res Clin Oncol. (2021) 147:275–85. doi: 10.1007/s00432-020-03408-2 33025281 PMC11801961

[3] Henschke CI Yankelevitz DF Libby DM Pasmantier MW Smith JP Miettinen OS . Survival of patients with stage I lung cancer detected on CT screening. N Engl J Med. (2006) 355:1763–71. doi: 10.1056/NEJMoa060476 17065637

[4] Halder A Dey D Sadhu AK . Lung nodule detection from feature engineering to deep learning in thoracic CT images: a comprehensive review. J Digit Imaging. (2020) 33:655–77. doi: 10.1007/s10278-020-00320-6 31997045 PMC7256172

[5] MacMahon H Naidich DP Goo JM Lee KS Leung ANC Mayo JR . Guidelines for management of incidental pulmonary nodules detected on CT images: From the Fleischner Society 2017. Radiology. (2017) 284:228–43. doi: 10.1148/radiol.2017161659 28240562

[6] Yip SS Aerts HJ . Applications and limitations of radiomics. Phys Med Biol. (2016) 61:R150–66. doi: 10.1088/0031-9155/61/13/R150 27269645 PMC4927328

[7] Rizzo S Botta F Raimondi S Origgi D Fanciullo C Morganti AG . Radiomics: the facts and the challenges of image analysis. Eur Radiol Exp. (2018) 2:36. doi: 10.1186/s41747-018-0068-z 30426318 PMC6234198

[8] Zwirewich CV Vedal S Miller RR Müller NL . Solitary pulmonary nodule: high-resolution CT and radiologic-pathologic correlation. Radiology. (1991) 179:469–76. doi: 10.1148/radiology.179.2.2014294 2014294

[9] Dennie C Thornhill R Sethi-Virmani V Souza CA Bayanati H Gupta A . Role of quantitative computed tomography texture analysis in the differentiation of primary lung cancer and granulomatous nodules. Quant Imaging Med Surg. (2016) 6:6–15. doi: 10.3978/j.issn.2223-4292.2016.02.01 26981450 PMC4775240

[10] Wang H Guo XH Jia ZW Li HK Liang ZG Li KC . Multilevel binomial logistic prediction model for Malignant pulmonary nodules based on texture features of CT image. Eur J Radiol. (2010) 74:124–9. doi: 10.1016/j.ejrad.2009.01.024 19261415

[11] Digumarthy SR Padole AM Lo Gullo R Singh R Shepard JO Kalra MK . CT texture analysis of histologically proven benign and Malignant lung lesions. Med (Baltimore). (2018) 97:e11172. doi: 10.1097/MD.0000000000011172 29952966 PMC6039644

[12] Shen J Du H Wang Y Du L Yang D Wang L . A novel nomogram model combining CT texture features and urine energy metabolism to differentiate single benign from Malignant pulmonary nodule. Front Oncol. (2022) 12:1035307. doi: 10.3389/fonc.2022.1035307 36591441 PMC9798090

[13] Choe J Lee SM Do KH Lee G Lee JG Lee SM . Deep learning-based image conversion of CT reconstruction kernels improves radiomics reproducibility for pulmonary nodules or masses. Radiology. (2019) 292:365–73. doi: 10.1148/radiol.2019181960 31210613

[14] Kanal KM Butler PF Sengupta D Bhargavan-Chatfield M Coombs LP Morin RL . U.S. diagnostic reference levels and achievable doses for 10 adult CT examinations. Radiology. (2017) 284:120–33. doi: 10.1148/radiol.2017161911 28221093

[15] Wang Z Zhu W Yang M Du H Zhou F Song N . Air bronchogram on chest CT in radiological pure-solid appearance lung cancer: Correlation analysis with genetic pathological features and survival outcomes. Eur J Radiol. (2023) 169:111194. doi: 10.1016/j.ejrad.2023.111194 37976762

[16] Li M Li X Guo Y Miao Z Liu X Guo S . Development and assessment of an individualized nomogram to predict colorectal cancer liver metastases. Quant Imaging Med Surg. (2020) 10:397–414. doi: 10.21037/qims.2019.12.16 32190566 PMC7063284

[17] Koo TK Li MY . A guideline of selecting and reporting intraclass correlation coefficients for reliability research. J Chiropr Med. (2016) 15:155–63. doi: 10.1016/j.jcm.2016.02.012 27330520 PMC4913118

[18] Haralick RM Shanmugam K Dinstein I . Textural features for image classification. IEEE Trans Syst Man Cybern. (1973) SMC-3:610–21. doi: 10.1109/TSMC.1973.430931425079929

[19] DeLong ER DeLong DM Clarke-Pearson DL . Comparing the areas under two or more correlated receiver operating characteristic curves: a nonparametric approach. Biometrics. (1988) 44:837–45. doi: 10.2307/2531595 3203132

[20] Collins GS Moons KGM Dhiman P Riley RD Beam AL Van Calster B . TRIPOD+AI statement: updated guidance for reporting clinical prediction models that use regression or machine learning methods. BMJ. (2024) 385:e078378. doi: 10.1136/bmj-2023-078378 38626948 PMC11019967

[21] Aerts HJWL Rios Velazquez E Leijenaar RTH Parmar C Grossmann P Carvalho S . Decoding tumour phenotype by noninvasive imaging using a quantitative radiomics approach. Nat Commun. (2014) 5:4006. doi: 10.1038/ncomms5006 24892406 PMC4059926

[22] Lu J Song C Xu H Fan J Liu K Chen J . Longitudinal variability of CT imaging features for predicting pulmonary nodule invasiveness: A multicenter study. Chin J Cancer Res. (2025) 37:781–95. doi: 10.21147/j.issn.1000-9604.2025.05.10 41229981 PMC12603625

[23] Pianigiani T Cameli P Di Lorenzo M Bergantini L Bargagli E . Longitudinal multimodal assessment of pulmonary nodules in fibrosing interstitial lung diseases: a retrospective study. Lung. (2026) 204:11. doi: 10.1007/s00408-026-00877-z 41801486

